# Genetic Analysis of Mu and Kappa Opioid Receptor and COMT Enzyme in Cancer Pain Tunisian Patients Under Opioid Treatment

**Published:** 2017-12

**Authors:** Imen CHATTI, Jean-Baptiste WOILLARD, Amira MILI, Isabelle CREVEAUX, Ilhem BEN CHARFEDDINE, Jihène FEKI, Sarah LANGLAIS, Leila BEN FATMA, Ali SAAD, Moez GRIBAA, Frédéric LIBERT

**Affiliations:** 1. Laboratory of Human Cytogenetics, Molecular Genetics and Reproductive Biology, Farhat Hached Hospital, Sousse, Tunisia; 2. U850 INSERM, University of Limoges, CHU Limoges, FHU SUPORT, Limoges, France; 3. Laboratoire de Biochimie Médicale, Faculté de Médecine, Clermont-Ferrand, France; 4. Service d’Oncologie Médicale et Service de Chirurgie Générale, CHU Habib Bourguiba, Université de Sfax, Sfax, Tunisia; 5. Dept. of Medical Oncology, CHU Farhat Hached, Sousse, Tunisia; 6. Laboratoire de Pharmacologie et Toxicologie, CHU G. Montpied, Clermont-Ferrand, France; 7. Inserm, U1107 NEURO-DOL, Clermont Université, 63001 Clermont-Ferrand, France

**Keywords:** Polymorphism, *OPRM1*, *OPRK1*, *COMT*, Cancer pain, Genetic heterogeneity

## Abstract

**Background:**

Pain and its opioid treatments are complex measurable traits. Responses to morphine in terms of pain control is likely to be determined by many factors, including the underlying pain sensitivity of the patient, along with nature and extent of the painful process, concomitant medications, genetic and other clinical and environmental factors. This study investigated genetic polymorphisms implicated in the inter-individual pain response variability to opioid treatment in the Tunisian population.

**Methods:**

This prospective association study investigated seven variations in the *OPRM1*, *OPRK1* and *COMT* gene, which encode Mu and KAPPA opioid receptors, and Catechol-O-methyltransferase enzyme respectively, in a cohort of 129 Tunisian cancer pain patients under oral morphine treatment. Genotyping was performed by simple probe probes on Light Cyler for rs17174629, rs1799972, rs1799971, rs1051659, rs1051660 and rs4680 and by PCR assay for the indel in the promoter region of OPRK1 (rs35566036). A statistical associations study between dose (continuous), dose escalation (yes/no) and SNP or haplotypes were investigated using linear multiple regressions and logistic regressions respectively adjusted on metastases and pain covariates in the R software.

**Results:**

We detected significant association of the rs1051660 adjusted on metastasis and pain (*P*=0.02), no other association has been detected between the 7 polymorphisms screened and the dose of morphine needed for pain relief.

**Conclusion:**

This can be explained by the strong genetic heterogeneity in the cosmopolitan areas where our patients were recruited for this study, compared to more homegenous population recruited in other studies.

## Introduction

Opioid therapy is a mainstay in acute and chronic pain management especially for cancer-related pain (1). Genetic causes for inter-individual variability of the clinical response to opioids have been proved for several years.

Pharmacological studies have defined three sub-types of morphinic receptors: mu, kappa, and delta. Many SNPs in morphinic pathway genes have been implicated in the inter-individual pain response variability to opioid treatment.

As the most important target of morphine, the μ-opioid receptor (MOR) mediates most of the opioid actions, including analgesia, tolerance, and reward. Polymorphisms of the *OPRM1*gene, coding for the MOR, are the primary candidates for the genetic influence on the efficacy of opioids, especially the 118 A>G variant (db SNP Accession No. rs1799971). Other polymorphisms in this gene have been investigated for possible association with alcohol or opioid abuse such as the 17C>T variant (db SNP Accession No. rs1799972), and the −1320 A>G variant (db SNP Accession No. rs17174629). GG genotype patients for the 118 A>G SNP need more morphine to obtain pain control. The 118A>G variant has been shown to alter the μ-opioid receptor signaling and/or expression in the human brain (2–5). A significant association between the 17C>T polymorphism and drug and alcohol dependency have been reported, while other studies have failed to do so (6, 7).

The human *OPRK1* gene encodes the KAPPA opioid receptor (KOR) which has a functional interaction with MOR activation pathway(8). The KOR, when activated by the endogenous ligand dynorphin A1-17, plays a modulatory role in opioid, cocaine and other rewarding stimuli, presumably through modulation of basal and drug-induced dopaminergic tone(9). Variations in *OPRK1* (especially the SNP 36 G>T) were associated with alcohol and heroin dependence. Non-coding *OPRK1* variations (such as the INDEL rs 35566036) have been associated with opioid addiction (10, 11).

Catechol-O-methyltransferase (COMT) is partly responsible for catecholamine catabolism, especially in the nervous system. This SNP was previously associated with addiction. The *COMT* gene 158A>G polymorphism (db SNP Accession No. rs4680) in combination with *OPRM1* 118A>G variant has been associated with morphine dosing requirements in adults (5).

The objective of this study was to determine the possible association of variations in the *OPRM1, OPRK1* and *COMT* genes with morphine dosing for opioid therapy in Tunisians cancer patients.

## Methods

Overall, 129 Tunisian cancer patients from Farhat Hached Hospital (Sousse, Tunisia), Habib Bourguiba Hospital (Sfax, Tunisia) and Salah Azaiaz Institut (Tunis, Tunisia), aged between 17 and 65 yr with normal urinary and hepatic function tests and with no history of morphine use, were included in this prospective association study. They were treated for at least three days with a stable oral morphine dose. Clinical characteristics were collected for each patient. They were divided into two groups; first group comprising patients relieved after the administration of the initial dose of morphine (60 mg/d), and second group with patients needing a dose escalation for pain relief (from 80 mg/d to 120 mg/d). All participants provided an informed consent and this study was approved by the local Ethics Committee.

### DNA extraction

All fresh blood was collected in EDTA tubes, and DNA was extracted using the FlexiGene DNA isolation Kit in compliance with the manufacturer’s protocol (Qiagen GmbH, Germany).

### Genotyping INDEL Genotyping

The *Indel* in the promoter region of the *KAPPA* gene was genotyped by polymerase chain reaction (PCR) using MultiGene^TM^Mini thermocycler (Labnet International, Inc) with selective primers designed with Primer 3 software (V. 0.4.0): forward 5′ GAGGGCTTCTCAATGCTCTG 3′ and reverse5′ GGATTTGACCACCAGCGT 3′ which should amplify a wild-type 1166 bp and/or deleted 336 bp fragments. The following conditions were selected for PCR in a 25-μl final volume: 100 ng DNA, 1 U Taq polymerase (Invitrogen), 2 mM MgCl_2_ and 20 pmol primers. Amplification was carried out using an initial denaturing cycle at 94 °C for 5 min and subsequent 35 cycles as follows: denaturation 30 sec at 94 °C, annealing 30 sec at 60 °C and extension 30 sec at 72 °C. PCR products (10 μl per lane) were separated on 2% agarose gel and stained with ethidium bromide (0.5 μg/ml). The gel was subject to ultraviolet lights and photographed.

### SNP Genotyping with “Simple Probe” Probes

SNPs probes for polymorphisms in the *Mu*, *Kappa* and *COMT* genes were obtained from TIB MOLBIOL (Berlin, Germany). LightCycler-FastStart DNA Master Hybridization Probes kit and the LightCycler 2.0TM were from Roche Diagnostics (Mannheim, Germany).

DNA samples were amplified in 20μl volume reactions containing 1 μl Reagent Mix, 2 μl Fast Start DNA Master, 1,6 μl Mg Cl2 (25 mM) and 2 μl DNA ( 25 ng/μl). The Light cycler 480 2.0 programming is shown in Table 1.

**Table 1:** The Light cycler 480 2.0 programming for SNP detection

**Program**	**Denaturation**		**Cycling**			**Melting**		**Cooling**
*Parameter*								
Analysis Mode	None	Quantification				Melting Curves		None
Cycles	1		45			1		1
Segment	1	1	2	3	1	2	3	
Target [°C]	95	95	60	72				
Hold [hh :mm :ss]	00 :10 :00	00 :00 :10	00 :00 :10	00 :00 :15	00 :00 :20	00 :00 :20	00:00 :00	00 :00 :30
Ramp Rate [°C/s]	20.0	20.0	20.0	20.0	20.0	20.0	0.2	20.0
Acquisition Mode	None	None	None	None	None	None	Continu	None

For SNP analysis, the LightCycler Instrument monitors the melting behavior of the Simple Probes. By measuring the fluorescence, the instrument can detect melting of the probe-target hybrids as the temperature increases. The more stable the hybridization between SimpleProbe and target sequence, the higher the melting temperature. Mutations like SNPs weaken the stability of SimpleProbe binding.

In each sample, signal fluorescence clearly decreases as temperature increases, and curves show the melting temperature of each sample as a peak.

### Statistical analysis

The statistical analyses were performed using R software version 2.15.1 (R foundation for statistical computing, http://www.r-project.org). Patient’s characteristics were compared between groups using a chi-squared test with Yates correction. Deviations from Hardy–Weinberg equilibrium were assessed using the Hardy–Weinberg exact test with the package “SNPassoc”. For the MOR gene, the extent of disequilibrium was investigated and expressed in terms of D′ and r^2^. Associations between dose (continuous), dose escalation (yes/no) and SNP or haplotypes were investigated using linear multiple regressions and logistic regressions respectively (SNPassoc). Covariates investigated were the presence of metastasis, pain covariates (bones, visceral and headaches), age and sex. For association analyses of SNPs and haplotypes, the most frequent allele was considered as the reference. An additive genetic model was chosen for the regressions analyses.

## Results

The characteristics of the study population are presented in Table 2. The Mean age ±SD of the patients was 46± 19 yr.

**Table 2:** Patients characteristics for total study population

***Population characteristics***		**Control group (group 1)**		**Group2**		**P value**
		***Number (n)***	***Percentage (%)***	***Number (n)***	***Percentage (%)***	
**Age (yr)**	18 – 25	6	8.1	6	10.9	0.4426
	26 – 45	26	35.1	24	43.63	
	46 – 65	42	56	25	45.45	
**Sex**	Man	17	22.9	46	83.6	<0.0001
	Woman	57	77	9	16.3	
**Cancer type**	ORL	7	9.4	4	7.27	0.7048
	Lang	5	6.75	3	5.45	
	Digestive	19	25.6	16	29	
	Urogenital	21	28.3	10	18.1	
	Bones	7	9.45	10	18.1	
	Mammary	13	17.56	11	20	
	Haematological	2	2.7	1	1.8	
**Metastases**	No metastasis	37	50	18	32.7	0.0051
	Bone metastasis	12	16.2	23	41.8	
	Others	25	33.7	14	25.4	
**Pain**	Headaches	5	6.75	0	0	0.0002
	Bones	3	4	15	27.2	
	Visceral	66	89.1	40	72.7	

The minimum and maximum doses for pain relief were 10 mg/ml and 100 mg/ml twice a day respectively. The average dose ±SD was 40.3 ± 18.6 mg/ml.

### Genotype and haplotype distribution

All genotypes were in Hardy-Weinberg equilibrium. Genotype frequency data are detailed in Table 3. A strong linkage disequilibrium was observed between the OPRM1 SNPs rs17174629/rs1799972: D’=0.99, r^2^=0.92; rs17174629/rs1799971: D’=0.68, r^2^=0.28; rs1799972/rs1799971: D’=0.67, r^2^=0.30. The 2 haplotypes that occurred more frequently were AAC (85%) and AGC (12%), the 2 others (AAT and GAT) occurred in less than 3% of the samples.

**Table 3:** Frequency and distribution of the studied polymorphisms

***Gene/SNP***	***Genotype***	***Genotype [% (n)]***			***Allele [%]***	
		**AA**	**Aa**	**aa**	**A**	**a**
OPRM1						
rs17174629	−1320 A>G	95.3 (123)	4.7 (6)	0.0 (0)	97.7	2.3
rs1799972	17 C>T	94.6 (122)	5.4 (7)	0.0 (0)	97.3	2.7
rs1799971	118 A>G	76 (98)	24 (31)	0.0 (0)	88	12
OPRK1						
rs1051659	15 C>T	100 (129)	0.0 (0)	0.0 (0)	100	0
rs1051660	36 G>T	84.5 (109)	13.2 (17)	2.3 (3)	91.1	8.9
rs35566036	INDEL	19 .4 (25)	37.2 (48)	43.4 (56)	Del : 60.5	INS : 39.5
COMT						
rs4680	158 A>G	32.6 (42)	44.2 (57)	23.3 (30)	54.7	45.3

No heterozygous or homozygous mutant for rs1051659 in OPRK1 is found in this study. This fact did not allow statistical analysis for this variant.

### Influence of genetic variables on morphine dose requirement

Multivariate analysis showed no significant association between genetic polymorphisms studied as SNP or haplotype adjusted and dose requirement (univariate analysis of genetic variable Table 4). Only the pain was significantly associated with morphine dose with an increased dose requirement for bone pain (bone vs. visceral pain, β±standard deviation (SD): 11.4±4.6, *P*=0.0151).

**Table 4:** Univariate analysis of genetic covariate on dose requirement

***Gene***	***Variable***	***Category***	***difference***	***95% Confidence Interval***	**P *value***
OPRM1	rs17174629	G vs A	−7.98	−22.82 ; 6.86	0.2939
	rs1799971	G vs A	0.20	−7.23 ; 7.62	0.9585
	rs1799972	T vs C	−6.86	−20.64 ; 6.91	0.3305
	haplotype	AGC vs AAC	0.74	−6.86 ;8.34	0.8490
	rs17174629/ rs1799971/ rs1799972				
OPRK1	Rs1051660	T vs G	−3.00	−10.13;4.13	0.4116
COMT	rs4680	A vs G	−2.10	−6.33; 2.14	0.3337

### Influence of genetic variables on morphine dose escalation

Multivariate analysis showed that the 36G>T SNP in the OPRK1 gene (rs1051660) (T vs*.* G OR=0.33 (0.10–0.87), *P*=0.03790) and pain (bones vs visceral OR=6.54 (2.06–26.68), *P*=0.00323) were significantly associated to morphine dose escalation (univariate analysis of genetic variable Table 5).

**Table 5:** Univariate analysis of genetic covariate on dose escalation

***Gene***	***Variable***	***Category***	***OR***	***95% Confidence Interval***	**P *value***
**OPRM1**	rs17174629	G vs A	0.19	0.02 ; 1.80	0.0984
	rs1799971	G vs A	1.92	0.79 ;4.66	0.1490
	rs1799972	T vs C	0.44	0.08 ;2.55	0.3441
	haplotype rs17174629/ rs1799971/ rs1799972	AGC vs AAC	1.83	0.75;4.48	0.1890
**OPRK1**	rs35566036	T vs A	0.84	0.51 ; 1.41	0.5159
	Rs1051660	T vs G	0.34	0.12 ;0.98	0.0278
**COMT**	rs4680	A vs G	0.76	0.45 ;1.27	0.2928

## Discussion

We screened seven SNPs in Mu opioid receptor, Kappa opioid receptor and COMT genes for genetic vulnerability to morphine dose requirement in case of cancer pain patients.

For the first time, we performed a multivariate statistical analysis. This allowed exploring the clinical and the genetic factors associated with the variability of morphine treatments and finding for the first time, an association between one SNP in the *KAPPA* gene and clinical characteristics.

The response to treatment of pain is of great inter-individual variability. This variability is due to social, environmental and probably genetic factors (12). Opioids are a basic treatment in the management of pain due to cancer. They target opioid receptors and their genes have several polymorphisms, which can influence the response to treatment (13). Therefore, genes coding for the opioid receptors should be candidates for an exploratory genetic association study.

In the present study, seven SNPs were screened in Mu opioid receptor, Kappa opioid receptor and COMT genes for genetic vulnerability to morphine dose requirement in case of cancer pain patients. Several studies evaluated the impact of the single nucleotide polymorphisms in the *OPRM1*, the *OPRK1* and the *COMT* genes on the variation in morphine doses for analgesia (13, 14).

Concerning the μ-opioid receptor gene, we studied three variants −1320A>G, 118A>G and 17C>T. The −1320A>G variant has a frequency of 2.3%, which is high compared with the 0.6% and 0.21% reported in Caucasian population but lower than the 9.1% in African American(10, 15). This variant, taken separately or in combination with the others or haplotypes, has no impact on the morphine dose needed in Tunisian cancer patients. In 2003, any association was found between opioid addiction and the −1320 variant (15). Haplotype analyses revealed that this variation belongs to a characteristic pattern of sequence variants associated with substance dependence (2). Our study showed that the frequency of the G allele in the 118A>G variant is 12% (Table 2) and there was no association with the morphine dose needed. This frequency is similar to Caucasian patients (10% to 14%) with discordant study association results and lowers than the 24.5% found in the Taiwanese patients, in where an association was found (3, 16, 17).

The influence of eight single nucleotide polymorphisms was investigated within the μ-opioid receptor promoter and evaluated the frequencies of the relevant SNPs in 700 patients on opioid medication (10). Nevertheless, and because of the low frequency of some variations (in comparison to African Americans’), an association analysis of opioid requirements was not feasible. The contribution of 118A>G SNP was evaluated for variability in responses to morphine treatment in 162 Caucasian cancer patients (16). There was no association.

In the present study, the frequency of the T allele of the 17C>T polymorphism (rs1799972) in *OPRM1* gene was 2.7% (Table 2). No significant association has been detected of this SNP related to the dose of morphine. The frequency of the minor T allele varies from 1% in white and Eastern Asian populations to 15%–20% in African Americans and northern Indians ones (18–21). In 2012, this polymorphism was studied in African American women. They a highly significant association of the T homozygote genotype with the alcohol, cocaine and tobacco consumption was found. No significant association has been found between the 17C>T polymorphism and the opiate use (22).

We investigated an INDEL in the promoter region (rs35566036) of *OPRK1* gene and found a frequency 39.5%. However, no association of this variant with the dose of morphine required in our population was proved. The indel allele was the minor one, with a frequency of 28% in European American population. This indel was associated with alcohol dependence and other illicit drugs (23).

In this same *OPRK1* gene, we also studied two SNPs in the exon 2 (15C>T and 36G>T, rs1051659 and rs1051660 respectively). The 15C>T did not show any heterozygous or homozygous mutant. For the 36G>T variant, the minor T allele has a frequency of 8.9% and was associated with bone metastasis in Tunisian cancer pain patients (*P*=0.02783). This frequency is similar to the 8.2% reported in HapMap Data Base. Interestingly, there was no published report regarding the association between metastasis and this polymorphism. This variant was significantly associated with body weight in a cohort of Taiwan methadone maintenance treatment (MMT). The bone or joint ache score was significantly associated with others SNPs located at intron 3 and exon 4, found to be associated with the frequency and amount of alcohol use in MMT patients in Taiwan (24).

In the Indian population, *OPRK1* polymorphisms (rs16918875, rs702764, and rs963549) were studied and their interaction with A118 G of the *OPRM1* gene. They found the *OPRM1* risk allele G was invariably present suggesting an important contribution of *OPRM1* in the digenic inheritance for addiction (8).

The *COMT* gene encodes for the catechol-o-methyltransferase, which is a key enzyme that metabolizes catecholamine in the nervous system. Only the 158G>A variant has been studied in different ways, such as in combination with *OPRM1* polymorphisms in pain and pain-related phenotypes and in morphine pain relief (5, 17, 25, 26), with conflicting conclusions. The frequency of G variant in our studied population is 12%. Our results found no association between the *OPRM1* and the *COMT* gene-dose effect variants. The AA genotype in the *COMT* gene was associated with a decrease in the μ-opioid reaction to pain and increase its ligation capability to the receptor (27). A gene-dose effect on univariate analyses, with carriers of *COMT* GG allele (28). A heterozygous for OPRM1 and COMT SNP, patients have a decreased analgesic effect with morphine and increased pain intensity (29). The A allele for the COMT variant makes the protein less stable with different pain reaction and opioid doses requirements variations between individuals. In fact, AA variant of the COMT rs4680 single nucleotide polymorphism consumed 36% (95% confidence interval, 31%–41%) more opioids than patients homozygous for the GG variant (*P*=0.009) in postoperative nephrectomy patients (30).

Our study was the first in Tunisia to investigate the joint effects of polymorphisms in Mu and KAPPA opioid receptors genes and the *COMT* gene in the clinical efficacy of morphine for cancer patients. An association between the rs1051660 in the *OPRK1* and bone metastasis in cancer patients was found in this investigation. No other significant association has been found. The integration of other polymorphisms in other opiate receptor genes or in genes involved in muopioid receptors signaling genes may be investigated. However, this lack of association can be explained by the genetic characteristics of our population. Indeed, the Tunisian population, including Sousse, where most of our patients were recruited, shows a high *heterogeneity* (31). This might be due to the large influence of successive migration waves in this region. Population heterogeneity has to be taken into account for genetic studies, and especially in pharmacogenetics. By the way, the heterogeneity–induced variability makes it more difficult to reach significant levels.

## Conclusion

Pain is a complex human trait, which requires the interaction of multiple genes, each with a small individual effect combined with environmental factors. All these may influence the efficacy of opioid treatments, especially in Cancer. Moreover, diversity of cancers’ characteristics, of pain sensitivity and of therapeutic response may be factors increasing the difficulty to highlight genetic factors that may be linked to pain variation or to the effectiveness of drugs.

An association between the 36G>T SNP and the bone metastasis has been found. No other association has been detected in other studies for other populations. This result can be explained by the strong genetic heterogeneity of our population. The impact of such genetic heterogeneity will have to be taken into account for further association studies and pharmacogenetic studies, especially when these studies are performed in regions with a rich migration history.

## Ethical considerations

Ethical issues (Including plagiarism, informed consent, misconduct, data fabrication and/or falsification, double publication and/or submission, redundancy, etc.) have been completely observed by the authors.
